# iDLDDG: predicting protein stability changes from missense mutations in DNA-binding proteins using integrated deep learning features

**DOI:** 10.1093/bib/bbag050

**Published:** 2026-02-13

**Authors:** Xuan Yu, Fang Ge, Dong-Jun Yu, Zhaohong Deng

**Affiliations:** Department of Computer Science, City University of Hong Kong, 83 Tat Chee Ave, Kowloon Tong, Hong Kong SAR(HKG), 999077, China; State Key Laboratory of Flexible Electronics (LoFE) & Institute of Advanced Materials (IAM), Nanjing University of Posts & Telecommunications, 9 Wenyuan Road, Nanjing, Jiangsu Province, 210023, China; School of Computer Science and Engineering, Nanjing University of Science and Technology, 200 Xiaolingwei, Nanjing, Jiangsu Province, 210094, China; School of Computer Science and Engineering, Nanjing University of Science and Technology, 200 Xiaolingwei, Nanjing, Jiangsu Province, 210094, China; School of Artificial Intelligence and Computer Science, Jiangnan University, 1800 Lihu Avenue, Wuxi , Jiangsu Province, 214122, China

**Keywords:** missense mutation, DNA-binding protein, protein–DNA interaction, bioinformatics, deep learning

## Abstract

To understand disease mechanisms and advance therapies, accurately predicting how missense mutations alter protein–DNA binding affinity is critical. Many existing models neglect the unique characteristics of missense mutations in both double-stranded DNA-binding proteins (DSBs) and single-stranded DNA-binding proteins (SSBs). To address this issue, we constructed a comprehensive dataset from diverse sources. By leveraging sequence-based embeddings from pretrained protein language models including ESM2, ProtTrans, and ESM1v, we developed iDLDDG, a deep learning framework that integrates multi-scale structural and evolutionary information via a multi-channel architecture. To balance residue-wise information density against entropy, our entropy-based algorithm determined 181 residues as optimal for modeling biophysical constraints. This approach enhances predictive accuracy and computational efficiency, thereby supporting large-scale assessments of mutation effects in DNA-binding proteins. iDLDDG achieves state-of-the-art performance, with a 10-fold cross-validation PCC of 0.755 on MPD276 and 0.632 on independent test sets encompassing both DSBs and SSBs, significantly surpassing existing methods. By establishing the first computational framework that rigorously differentiates DSB and SSB mutation mechanisms, our work provides a foundation for high-accuracy prediction of pathological mutations in DNA-binding proteins.

## Introduction

Protein–DNA interactions (PDIs) are essential for key cellular processes such as DNA replication, repair, and recombination. Accurately predicting binding affinities is crucial for revealing biochemical mechanisms, including drug efficacy [1], antibody–antigen interactions, and early disease detection [2]. Binding affinity reflects the combined effects of intermolecular forces (electrostatic, van der Waals, hydrogen bonding, and hydrophobic interactions) and protein structural features [3–6]. Missense mutations perturb these elements, inducing significant binding free energy shifts [7]. ∆∆G quantifies mutation-induced binding free energy changes, serving as a key metric for protein stability prediction. While laboratory methods including isothermal titration calorimetry, electrophoretic mobility shift assays, and the systematic evolution of ligands through exponential enrichment yield precise ΔΔG measurements, their high cost and low throughput limit genomic-scale applications [8–10]. Consequently, computational approaches have gained prominence for their efficient ∆∆G prediction capabilities. Among the existing methods, FoldX uses high-resolution 3D structures to model and predict the impacts of mutations on binding energies, which is crucial for understanding mutation-induced ∆∆G changes [6, 11, 12]. Complementary to this, recent research has advanced our understanding of protein–nucleotide interactions, including PDIs and protein–RNA interactions (PRIs). For example, ProNIT provides thermodynamic data such as free energy and dissociation constants [13]. Building on this work, SAMPDI employs a modified molecular mechanics-based Poisson–Boltzmann surface area method to predict ∆∆G for MPDs [7]. PremPDI combines molecular mechanics with rapid side-chain optimization to assess mutation impacts [14]. For PRI studies, mCSM-NA integrates pharmacophore modeling with nucleic acid properties [15]. PEMPNI conducts systematic comparisons between mutations in DNA-binding proteins (MPDs) and RNA-binding proteins via specialized regression techniques and geometric energy features [16]. Together, these efforts establish a robust framework for analyzing how missense mutations affect protein–nucleotide interactions, offering insights into their structural and functional consequences.

Despite significant advances in PDI research, a critical distinction remains neglected: DNA-binding proteins encompass both double-stranded DNA-binding proteins (DSBs) and single-stranded DNA-binding proteins (SSBs) [17–19]. These two categories exhibit fundamental differences in functions, DNA structural specificity, and binding mechanisms. Prior studies demonstrate that DSBs and SSBs diverge in both sequence composition and structural attributes, driving the development of specialized classifiers [20, 21]. SSBs are essential for DNA replication, repair, and recombination processes, protecting single-stranded DNA and preventing the formation of secondary structures [22–24]. They bind with high affinity and can reposition along the strand [25]. In contrast, DSBs typically act as transcription factors or DNA repair proteins, maintaining genomic stability [26]. However, the existing models for predicting the effects of missense mutations on PDIs frequently utilize mixed datasets containing both DSBs and SSBs, potentially obscuring their fundamental functional and mechanistic distinctions. Moreover, existing models mainly rely on energy-based and structure-based features that require precise structural data and often fail to capture subtle molecular interactions [7, 16]. Recent advances in large-scale language models provide powerful sequence representations, such as ESM embeddings [27], with their ability to capture information that approximates atomic-resolution protein structure, offer a promising avenue to enhance predictive performance.

To enhance ∆∆G prediction for PDIs, we analyzed missense mutations in DSBs and SSBs separately, accounting for their distinct binding mechanisms. In this study, we constructed a comprehensive dataset (distinguishing between DSBs and SSBs) from multiple sources, ensuring accurate classification by referencing relevant studies. From these annotated proteins, we derived sequence embeddings via pretrained language models, including ESM2 [28], ESM1v [29], and ProtTrans [30], which capture intricate sequence embeddings. Then, by leveraging these embeddings, we constructed a deep learning model with a multi-channel input architecture, with ablation experiments demonstrating its structural imperative. Our model demonstrated superior performance when predicting ∆∆G for mutations in both DSBs and SSBs, outperforming the existing state-of-the-art methods.

## Materials and methods

### Benchmark dataset construction

**Figure 1:**
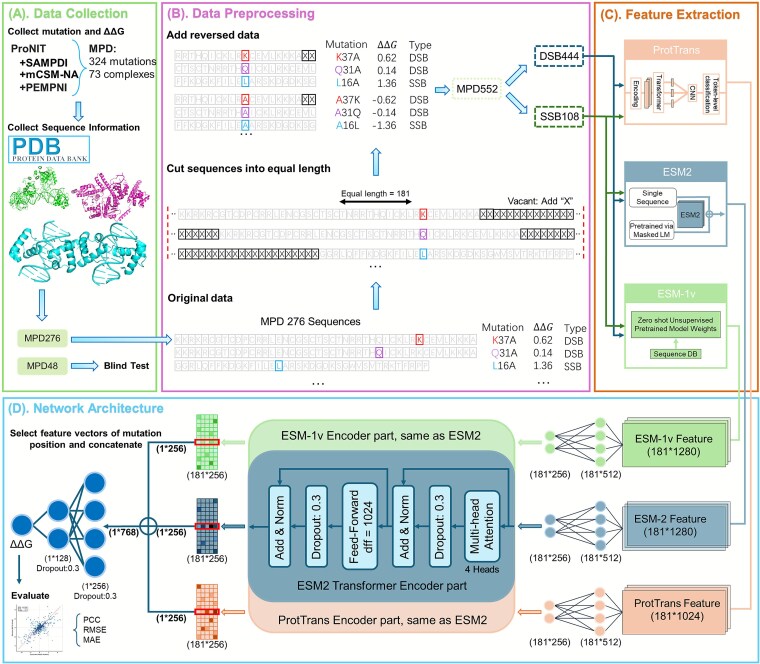
Overview of the iDLDDG workflow. (A) Mutation data collection; (B) mutation data preprocessing; (C) feature extraction; and (D) architecture of iDLDDG.

For comprehensive assessment of missense mutation effects on PDIs, we constructed a dataset capturing ∆∆G from 324 mutations across 73 complexes. This compilation integrates data from foundational studies, notably PEMPNI [16], mCSM-NA [15], and SAMPDI [7], which sourced data from dbAMEPNI [31] and ProNIT [13]. Each entry specifies the mutation position, the amino acid replacement, and the associated ∆∆G value. For precision, we corroborated these mutation sites with sequence records from the Protein Data Bank [32] (as depicted in Fig. 1A and B). Following the PEMPNI methodology, the dataset was partitioned into a training set, referred to as MPD276 (comprising 276 mutations from 53 complexes), and an independent test set, MPD48 (containing 48 mutations from 20 complexes). Notably, MPD48 was constructed to exclude any data used in the training of iDLDDG. Subsequently, the proteins in both sets were categorized into DSBs and SSBs via the ground truths provided by iPNHOT [22, 28]. The detailed information is provided in Tables S1 and S2.

Table S3 summarizes the statistical information of the benchmark datasets. MPD276 consists of DSB222 (222 mutations from 47 complexes) and SSB54 (54 mutations from 6 complexes), while MPD48 comprises IDSB35 (35 mutations from 15 complexes) and ISSB13 (13 mutations from 5 complexes). To ensure a more balanced dataset, we incorporated commonly used reverse mutations. This approach is grounded in the thermodynamic principle that the free energy change (∆∆G) for a mutation from wild type to mutant equals in magnitude but opposes in sign to that of its reverse mutation (i.e. ∆∆Gwildtype→mutant = ∆∆Gmutant→wildtype). This refinement is evident in the revised notations, such as MPD552 and DSB444.

### Feature representation

Precise numerical feature extraction from protein sequences is fundamental for predicting missense mutation effects. To address this need, we employ protein language models (PLMs) that generate context-aware residue embeddings. ProtTrans [30], ESM-2 [28], and ESM-1v [29] produce per-residue feature vectors capturing evolutionary constraints and structural contexts—particularly critical at mutation sites (as depicted in Fig. 1C). These embeddings enable deep learning models to decode hidden biophysical patterns unreachable by traditional methods.

ProtTrans [30], which was developed by using the UniRef and Big Fantastic Database dataset with up to 393 billion amino acids [33, 34], leverages 1024-dimensional embeddings per residue. For this study, we standardized the sequences to 181 residues centered on the mutation sites, producing a 181 × 1024 feature matrix per sample. These embeddings adeptly reflect protein structure and function constraints and have proven effective in tasks such as secondary structure prediction [35].

In contrast, ESM-2 [28], a transformer model with 650 million parameters, leverages 65 million distinct UniRef sequences [27, 36] through masked language modeling [37, 38]. This approach enables the model to discern complex sequence patterns, producing 1280-dimensional embeddings per residue. Applied to our 181-residue sequences, it produced a 181 × 1280 matrix, providing deep insights into the local environment of each mutation site.

Similarly, ESM-1v [29], which also features 650 million parameters, excels at zero-shot variant effect prediction. Trained on 98 million unannotated sequences [28, 39], it generated 1280-dimensional embeddings per residue, forming a 181 × 1280 matrix for each 181-residue sequence. This representation adeptly reflects the evolutionary and contextual details [29].

To ensure uniformity, we fixed the sequence length at 181 residues across all models, a decision guided by entropy analysis (please refer to Section 4.2 for more details), to optimize both the predictive performance and computational efficiency of the model. The sequences were aligned at the mutation site, with “X” padding applied when amino acids were insufficient, as shown in Fig. 1B.

### Cross-validation strategies

In this study, the dataset was split into 90% for training (with 10% of the training data randomly assigned as belonging to the validation set) and 10% for testing. The model was trained for 150 epochs, tracking validation performance to retain optimal checkpoints. Early stopping was set at 40 epochs to prevent overfitting. To provide a robust assessment, 10-fold cross-validation was used to divide the DSB444 dataset into ten equal parts, iteratively using nine parts for training and one rest part for testing, with averaged metrics ensuring reliable generalization estimates. Finally, independent datasets (MPD96 for the full model, IDSB70 for the DSB-focused model, and ISSB26 for the SSB-focused model) served as blind tests to evaluate the performance attained across diverse protein types.

### Evaluation metrics

To assess the predictive performance of the iDLDDG model, we employed multiple metrics to capture distinct performance aspects. First, the Pearson correlation coefficient (PCC) was used to measure the trend consistency between the predicted and true values; however, the PCC falls short when assessing absolute accuracy, as it may remain high despite consistent offsets in the predictions. Therefore, we incorporated complementary metrics: root mean square error (RMSE) amplifies large deviations, penalizing significant errors; mean absolute error (MAE) provides a robust average error metric, less sensitive to outliers. Together, these metrics provided a thorough performance evaluation of the iDLDDG model and enabled comparisons with the established methods. Specifically, the PCC tracks correlation, while the RMSE (kcal/mol) and MAE (kcal/mol) gauge the prediction precision on experimental data (detailed metric descriptions are available in Text S1).

## iDLDDG network architecture

In this study, we present iDLDDG, a novel computational model designed to predict ∆∆G alterations caused by missense mutations in PDIs (Fig. 1D**)**. The framework integrates three distinct feature matrices derived from PLMs (ESM-1v, ESM-2, and ProtTrans), all extracted from sequences centered on mutation sites.

Initially, the model processes feature through two fully connected layers, transforming them into 181 × 256 representations per peptide sequence. Positional encoding is then applied to preserve the sequence order. To model intricate contextual dependencies, we use three dedicated transformer encoders. Each encoder has two layers with multi-head attention (four heads) and a feedforward network, stabilized by dropout, layer normalization, and residual connections to ensure training stability and prevent overfitting.

Next, feature vectors at the mutation site are extracted from the output of each encoder and concatenated into a 768-dimensional vector. This combined vector is input into a neural network with two hidden layers (256 and 128 units) and an output layer that delivers the predicted ∆∆G. To optimize the model, we implemented the Pseudo–Huber loss function to assess the divergence between the predicted ${y}_{\mathrm{pred}}$ and true values ${y}_{\mathrm{true}}$, and it is defined as follows:


(1) \begin{equation*} {L}_{\delta }(a)={\delta}^2\left(\sqrt{1+{\left(\frac{y_{\mathrm{true}}-{y}_{\mathrm{pred}}}{\delta}\right)}^2}-1\right) \end{equation*}

where $\delta$ is a hyperparameter that balances the precision achieved for small errors with robustness to larger deviations. The multi-channel architecture in iDLDDG effectively integrates diverse protein representations, achieving high accuracy in predicting the effects of mutations on PDIs.

## Results and discussions

### Comparison between double-stranded DNA-binding proteins and single-stranded DNA-binding proteins from different viewpoints

**Figure 2:**
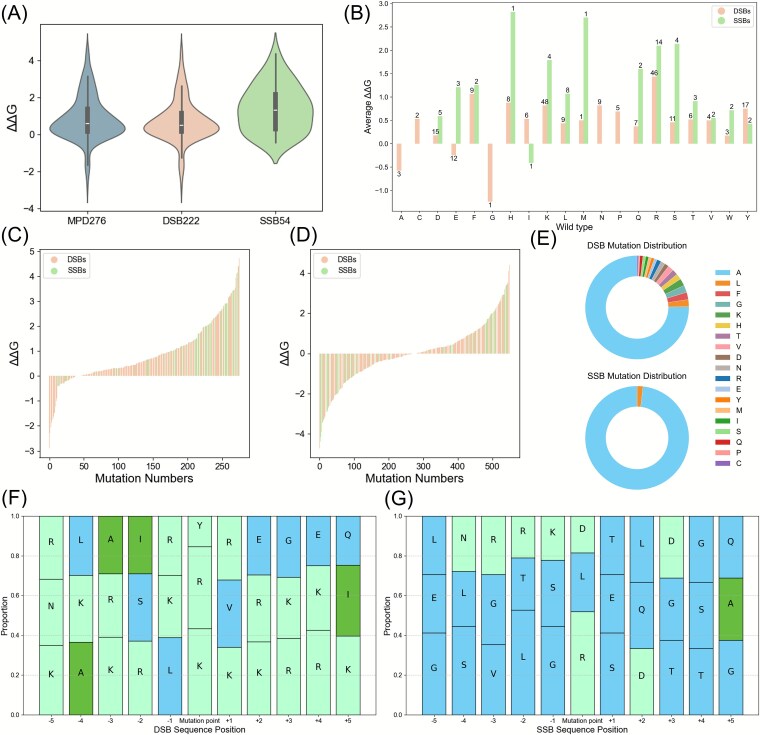
Comparative analysis of the DSB and SSB sub-datasets. (A) ΔΔG distributions for the MPD276, DSB222, and SSB54 datasets, revealing dataset-specific mutation effects; (B) mean ΔΔG values across 20 amino acid mutation types in both DSBs and SSBs, underscoring their differential impacts; (C) pre-augmentation ΔΔG distribution, skewed toward positive values, with a schematic depicting reverse augmentation (mutants revert to wild-type, ΔΔG negated); (D) post-augmentation ΔΔG distribution, equilibrated with positive and negative values, optimizing the applicability of deep learning; (E) distribution of mutated amino acids (F) top three amino acid frequencies within a ± 5 residue window for DSBs mutation sites; and (G) top three amino acid frequencies within a ± 5 residue window for SSB mutation sites.

In this section, a systematic comparison between DSBs and SSBs was conducted by using the DSB222 and SSB54 datasets, as depicted in Fig. 2. An analysis of the ΔΔG distributions (Fig. 2A) revealed that SSBs exhibit higher mean values and predominantly positive ΔΔG samples, whereas DSBs result in a more even split between positive and negative values. Furthermore, bar charts of the average ΔΔG across the 20 amino acids (Fig. 2B) revealed notable differences at mutation sites A, C, G, N, and P, where mutations occur only in DSBs and are absent in SSBs. Even at the most frequently mutated sites shared by both DSBs and SSBs, the average ΔΔG values differ substantially. For instance, at position R, the average ΔΔG for DSBs is lower than 1.5 kcal/mol, whereas that for SSBs exceeds 2 kcal/mol. Similar patterns can be observed at mutation sites K, L, and S.

To address the skewed ΔΔG distribution (Fig. 2C), a reverse augmentation approach was applied in which wild-type residues with negated ΔΔG values were reintroduced, yielding a balanced post-augmentation result (Fig. 2D) that was suitable for deep learning. From Fig. 2E, it can be observed that the distributions of mutated amino acids differ between DSBs and SSBs. In both cases, alanine (A) is the predominant mutated amino acid. However, for DSBs, ~25% of the mutations are distributed among other amino acids, indicating a more diverse mutation profile. In contrast, SSBs exhibit a much more concentrated pattern, with mutations occurring predominantly as alanine substitutions and only a very small fraction involving leucine (L). Additionally, the amino acid frequencies within a 10-residue window surrounding the mutation sites (Fig. 2F and G) exhibit clearly distinct patterns between DSB and SSB. Specifically, DSB-associated regions show a pronounced enrichment of positively charged residues such as arginine (R) and lysine (K), which is consistent with the strong electrostatic interactions required for binding double-stranded DNA. In contrast, SSB-associated regions are enriched in glycine (G), threonine (T), and leucine (L), residues that are commonly associated with increased conformational flexibility, facilitating interactions with single-stranded DNA. These differences highlight distinct sequence and physicochemical contexts around mutation sites in SSBs and DSBs, suggesting that the two protein classes exhibit fundamentally different binding and structural characteristics. Such heterogeneity in local sequence properties may lead to divergent feature representations, thereby motivating the use of separate models to better capture the mutation effects specific to each type of DNA-binding protein.

### Information entropy analysis for different input sequence lengths

When examining protein binding data, accounting for sequence length variations is essential. Typically, sequences are aligned around the missense mutation, trimmed to a uniform length (e.g. 41 AAs), and padded with “X” to standardize the input [40, 41]. Selecting an optimal length is critical, as it strongly affects model performance. Moreover, since protein sequences are analogous to character strings, they are naturally suited for processing with PLMs. To address this issue, we devised an algorithm that uses information entropy dynamics to determine the optimal sequence length.

**Algorithm 1:** Calculating entropies of aligned sequences with different cut lengths.

**Input**: Aligned sequence sets $\left\{{Seq}_1^L,\kern0.5em {Seq}_2^L,\dots{Seq}_n^L\ \right\}$ obtained under different cut lengths $L$ where $L\in \left\{{L}_1,{L}_2,\dots, {L}_k\right\}$. $n$ denotes the total number of sequences in a sequence set. $R$ denotes the set of amino acid residues including dummy amino acid “X.”

**Output**: Entropy array $H$, among which each element is the entropy corresponding sequentially to the cut sequence length $L$.

1: $H\leftarrow$ {} ▹ Initialize Entropy array

2: **for** each $L$ in $\left\{{L}_1,{L}_2,\dots, {L}_k\right\}$  **do**

3: Total_AAs $\leftarrow$  $L\ast n \ \\quad\triangleright$ Initialize total number of amino acids

4: All_chars $\leftarrow$ {} ▹ Save all peptides in one string

5: AA_counts $\leftarrow$ {} ▹ Save number of occurrences of given residue

6: **for each**$m$in $\left\{1,\dots, n\right\}$ do

7: All_chars.append(${Seq}_m^L$)

8: **end for**

9: **for each** residue **in**$R$  **do**

10: AA_counts.append (Number of occurrences of residue **in** All_chars)

11: **end for**

12: entropy $\leftarrow$ 0

13: **for each** element **in** AA_counts **do**

14: $p$  $\leftarrow$ element / Total_AAs

15: entropy $\leftarrow$ entropy - $p\ast{\mathit{\log}}_2(p)$

16: **end for**

17: $H$.append(entropy)

18: **end for**

19: **return**  $H.$

In this paper, we adjust the cut sequence length$L\in \left\{21,41,...,501\right\}$ with a step of 20, evaluating $n=552$ sequences in total. The increasing rate of valid amino acids defined as ${V}_{aa}$ is calculated by the following formula: 


$$ {V}_{aa}^k=\frac{P_{aa}^{k-1}{L}_{k-1}}{P_{aa}^k{L}_k}-1 $$

(2) \begin{align*} k\ge 2 \end{align*}

where ${P}_{aa}^k$ denotes the percentage of valid amino acids (exclude dummy padding “X”) in total amount of amino acids $n{L}_k$.

**Figure 3:**
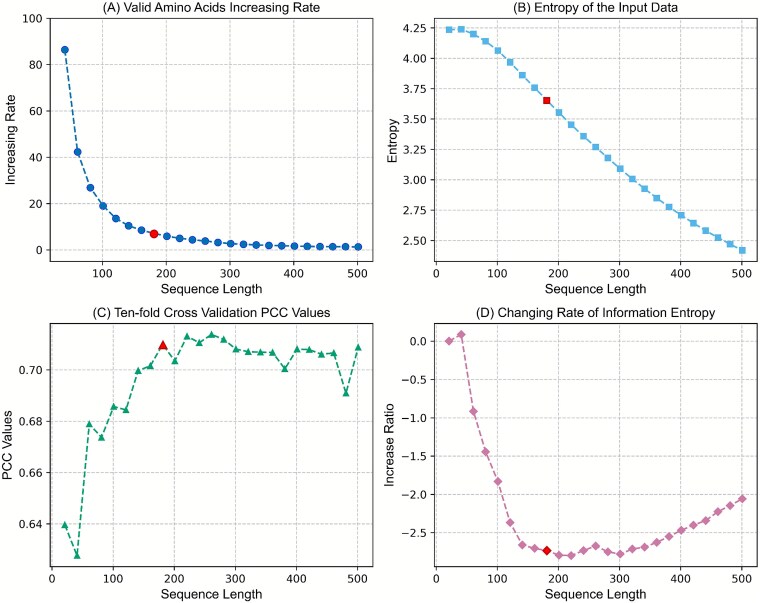
Analysis of the effects of different sequence lengths on information metrics with sequence lengths ranging from 21 to 501. (A) Valid amino acid increasing rate; (B) entropy; (C) 10-fold cross-validation PCC; and (D) changing rate of information entropy.

As shown in Fig. 3A, ${V}_{aa}$ decreases with increasing cut length of sequences, approaching zero by around 161 AAs. This suggests that beyond a certain threshold, additional length contributes minimal new information. The designed Algorithm 1 computes the information entropy of aligned sequence sets with different cut lengths. As shown in Fig. 3B, the entropy increases from 21 to 41 AAs before steadily decreasing. Finally, we evaluated the model performance using 10-fold cross-validation and averaged PCC scores (Fig. 3C). The results show that performance improves as entropies decrease. Notably, an exception occurs at 41 AAs, where the performance decreases with higher entropy, corresponding to a positive entropy change rate (Fig. 3D).

These results yield two key observations. First, predictive accuracy relies on adequate valid information, once that plateaus, performance gains cease. Second, high entropy may introduce noise, thus obscuring patterns, whereas excessively low entropy risks underfitting due to insufficient complexity. Consequently, we identify 181 amino acids as the optimal input length. This optimal point is marked by red dots in all four subfigures of Fig. 3, indicating the position where the model achieves its best performance. As shown in Fig. 3A, beyond this optimal point, the valid amino acid increasing rate approaches zero, suggesting that extending the sequence length introduces little additional informative content. Meanwhile, Fig. 3D demonstrates that the overall entropy of the input information decreases rapidly after this point, indicating diminishing information diversity. Furthermore, Fig. 3C shows that the 10-fold cross-validation PCC gradually stabilizes once the sequence length exceeds 181 residues. These observations indicate that further increasing the input length does not lead to substantial improvements in predictive performance, and therefore 181 residues are selected as the optimal sequence length. This approach refines our ability to predict the effects of mutations on DNA-binding proteins and establishes a versatile framework for broader protein dataset analyses.

### Performances achieved by iDLDDG on benchmark and separated datasets

To rigorously assess the ability of the iDLDDG model to predict the effects of missense mutations on DNA-binding proteins, we conducted 10-fold cross-validation on three distinct datasets (including MPD552, DSB444, and SSB108) by using uniform model parameters. The outcomes, detailed in Table 1, reveal varied performances across these sets. Specifically, on MPD552, iDLDDG attained an average PCC of 0.755, an MAE of 0.674, and an RMSE of 0.964 over 10-folds cross-validation. In contrast, training solely on DSB444 resulted in a diminished PCC of 0.681, a marginally elevated MAE of 0.694, and an RMSE of 1.031. Finally, SSB108 training yielded a notable PCC increase to 0.863, accompanied by increases in the MAE and RMSE to 0.764 and 0.996, respectively. These results indicate a PCC improvement of 0.108 for SSB108 and a decline of 0.074 for DSB444, underscoring the influence of dataset-specific traits. Since parameter optimization was not pursued, the performance remained suboptimal, suggesting that tailored adjustments could enhance the outcomes.

**Table 1:** Prediction performance achieved in 10-fold cross-validation experiments on benchmark datasets.

Datasets	PCC (kcal/mol)	MAE (kcal/mol)	RMSE (kcal/mol)
MPD552	0.755 ± 0.135	0.674 ± 0.149	0.964 ± 0.324
DSB444	0.681 ± 0.139	0.694 ± 0.161	1.031 ± 0.270
SSB108	0.863 ± 0.092	0.764 ± 0.319	0.996 ± 0.405

**Figure 4:**
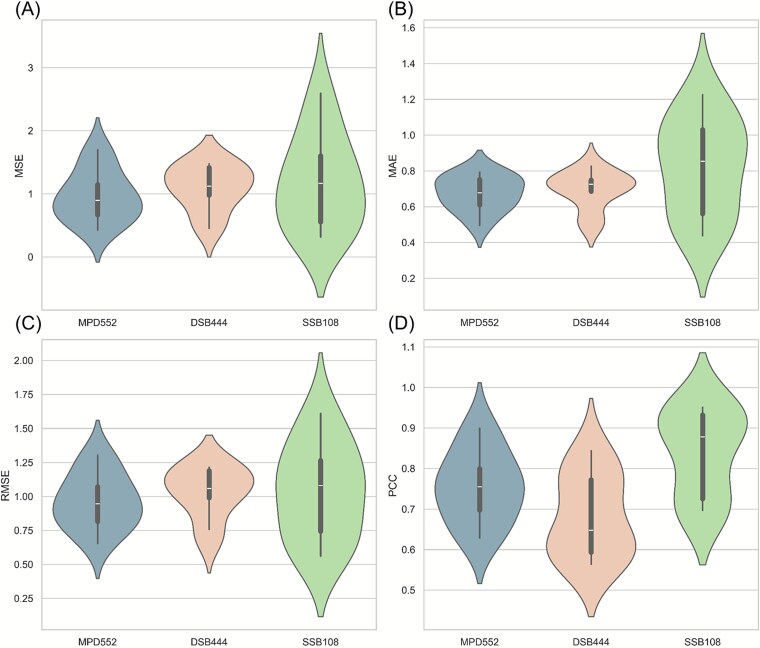
Performance evaluation of the iDLDDG model across different datasets (including MPD552, DSB444, and SSB108) via 10-fold cross-validation. (A) The MSE, (B) MAE, (C) RMSE, and (D) PCC.

The results in Fig. 4 further show that the models trained by MPD552 demonstrate balanced performance across all metrics. Although the PCC attained on DSB444 is lower, its MSE and RMSE demonstrate greater stability. In contrast, SSB108 shows a higher average PCC, but performance fluctuates significantly across different folds. This variability is likely due to the smaller dataset size, where suboptimal data in any fold can significantly impact the results. These observations affirm the promise of iDLDDG for DNA–protein interaction prediction yet highlight the necessity of refinement when addressing imbalanced or limited datasets.

### Ablation study

In our ablation study, we assessed the individual and combined contributions of the three feature sets (i.e. ESM1v, ESM2, and ProtTrans) to the predictive performance of our model. By training the model with different combinations of these features, we evaluated their impact by employing key metrics, namely, the PCC, MAE, and RMSE, with the results averaging over 10-fold cross-validation (Table 2).

**Table 2:** Prediction performances achieved in the feature ablation studies on the MPD552 dataset.

Feature Set	PCC (kcal/mol)	MAE (kcal/mol)	RMSE (kcal/mol)
ESM1v	0.731	0.667	1.027
ESM2	0.687	0.736	1.089
ProtTrans	0.708	0.751	1.079
ESM1v + ESM2	0.711	0.725	1.054
ESM1v + ProtTrans	0.713	0.712	1.052
ESM2 + ProtTrans	0.719	0.723	1.041
ESM1v + ESM2 + ProtTrans	0.755	0.674	0.964

The results reveal that the integration of all three feature sets yields the highest PCC (0.755) and the lowest RMSE (0.964), indicating a strong correlation between the predicted and actual values and the effective minimization of the prediction errors. Notably, while the MAE induced for this comprehensive combination is marginally higher (0.674) than that of ESM1v alone (0.667), this slight increase is outweighed by the substantial enhancements in the PCC and RMSE, thereby affirming the superiority of the full feature set.

Examining the pairwise combinations further elucidate the feature interactions. For instance, combining ESM1v and ESM2 yields a PCC of 0.711, lower than the 0.731 achieved by ESM1v alone. Pairing ESM2 with ProtTrans results in a PCC of 0.719, surpassing the individual performances of both ESM2 (0.687) and ProtTrans (0.708). This suggests a synergistic effect between ESM2 and ProtTrans. Integrating all three features leads to the most significant improvement in performance, underscoring the value of combining diverse data representations to optimize prediction accuracy in modeling the effects of missense mutations on DNA-binding proteins.

### Performance analysis of iDLDDG in cross-validation and independent tests

**Figure 5:**
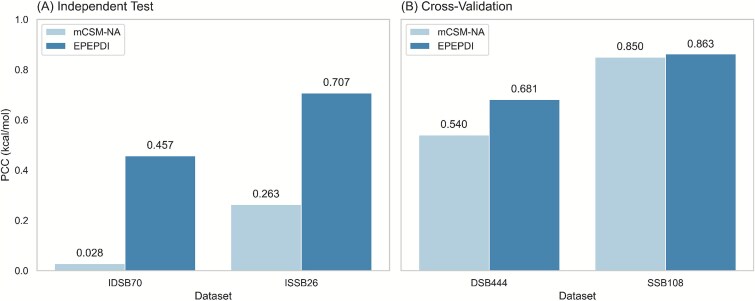
Comparisons of iDLDDG with mCSM-NA on (A) IDSB70 and ISSB26 (B) DSB444 and SSB108 datasets.

**Figure 6:**
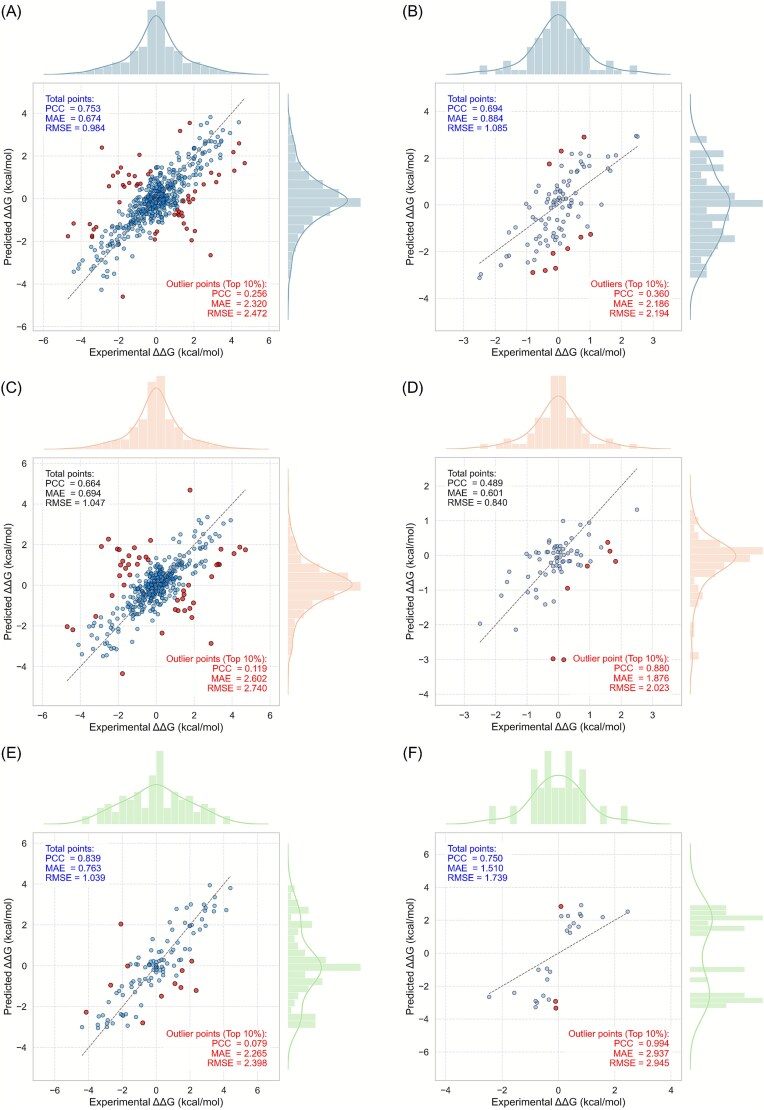
Scatter plots of different datasets, red points denote 10% outlier data. (A) MPD552 cross-validation, (B) MPD96 independent test, (C) DSB444 cross-validation, (D) IDSB70 independent test, (E) SSB108 cross-validation, and (F) ISSB26 independent test sets.

To evaluate the performance of iDLDDG, we compared it with established methods through cross-validation and independent testing. This analysis is based on data from Table 3 and Figs 5 and 6. The results in Fig. 6A, C, and  E were derived from the combined test set of each 10-fold cross-validation run, with a single PCC calculated for each run. This approach explains the slight discrepancy with the average PCC reported in Table 3. Figure 6B, D, and  F present the results for the independent test sets MPD96, IDSB70, and ISSB26, respectively. These results were generated using models that achieved the highest PCC during cross-validation on the MPD552, DSB444, and SSB108 datasets, correspondingly. This demonstrates the robustness of iDLDDG and its superior performance compared to other methods.

**Table 3:** Performance comparison with existing methods on the MPD552 and MPD96 datasets.

Validation Method and Dataset	Methods	PCC (kcal/mol)	RMSE (kcal/mol)
Cross-validation (MPD552)	iDLDDG	0.755	0.964
	PEMPNI	0.575	0.978
Independent Test Set (MPD96)	iDLDDG	0.632	0.942
	PEMPNI	0.550	0.670
	SAMPDI	0.424	0.705
	PremPDI	0.509	0.887
	mCSM-NA	0.016	1.259

#### Performance comparison on MPD552 and MPD96

As reported in Table 3, iDLDDG achieved an average PCC of 0.755 and an average RMSE of 0.964 during 10-fold cross-validation on the MPD552 dataset, surpassing PEMPNI (PCC = 0.575, RMSE = 0.978). These results suggest greater predictive precision and consistency for iDLDDG on training data. Figure 6A provides additional evidence, displaying a scatter plot of the MPD552 cross-validation results with metrics (PCC = 0.753, MAE = 0.674, RMSE = 0.883) that are consistent with those reported in Table 3. In terms of the scatter plots of the models trained via 10-fold cross-validation, the metrics were calculated by combining the results derived from each fold. Furthermore, for the scatter plots of the models evaluated with the independent test, the best-performing model was selected for plotting. As a result, the values in the scatter plots may slightly differ from the average metrics presented in Table 3. The corresponding histogram indicates that both the predicted and experimental values follow a near-normal distribution, mostly between −2 and 2 kcal/mol, reinforcing the reliability of the proposed model.

After conducting independent testing on the MPD96 dataset, Table 3 reveals the average PCC of 0.632 and RMSE of 0.942 yielded by iDLDDG, which indicate that the proposed model outperforms PEMPNI (PCC = 0.550, RMSE = 0.670), SAMPDI (PCC = 0.424, RMSE = 0.705), PremPDI (PCC = 0.509, RMSE = 0.887), and mCSM-NA (PCC = 0.016, RMSE = 1.259). Furthermore, the notably higher PCC of iDLDDG underscores its robust generalizability to unseen data. However, the lower RMSE of PEMPNI (0.670) indicates its strength in terms of limiting large prediction errors. On the independent MPD96 test set, iDLDDG achieved a PCC of 0.694, MAE of 0.884, and RMSE of 1.085 (Fig. 6B). These values differ slightly from the RMSE shown in Table 3, due to it shows the best model performance rather than average performance; however, they still affirm the overall superiority of iDLDDG. The detailed per-fold performance is summarized in Figs S1 and S2 for models trained on DSB444, and in Figs S3 and S4 for those trained on SSB108.

On the independent MPD96 test set, iDLDDG maintained a strong average PCC of 0.632, which consistently exceeded those of the other methods. Interestingly, its RMSE here is marginally lower than that in the cross-validation experiment, reflecting stable performance. Since the RMSE is sensitive to outliers, this metric highlights the capacity of the model to manage extreme values. For example, the distribution plot of the MPD276 benchmark dataset in Fig. 2 reveals outliers such as variants 2A0I_S3A (ΔΔG = 4.1 vs. series average 2.0) and 1AAY_R22A (ΔΔG = 3.54 vs. series average 1.19), far exceeding the dataset mean ΔΔG of 0.874. These anomalies emphasize the generalization strength of iDLDDG while explaining the variations in the RMSE.

#### Enhanced performance achieved via differential DNA-binder training

Building upon the core biophysical distinctions between SSBs and DSBs, we engineered a specialized training protocol for iDLDDG by using the DSB444 and SSB108 datasets. This structure-aware approach explicitly adapts to differential binding mechanics. When benchmarked against mCSM-NA using the independent test sets IDSB70 and ISSB26, the results presented in Fig. 5 reveal notable advantages in terms of the PCC. Figure 6 presents a diagnostic analysis of model behavior across different datasets using integrated scatter plots of predicted versus experimental values. In each plot, the red markers highlight the top 10% outliers, which are defined as mutations with the most extreme ΔΔG values within the corresponding dataset, including both highly positive and highly negative cases. These outliers provide a focused view of model performance on extreme-value predictions. Cross-validation results further demonstrate the clear superiority of iDLDDG. As shown in Fig. 5B, on the DSB444 dataset, iDLDDG achieves a PCC of 0.681, substantially outperforming mCSM-NA, which attains a PCC of 0.540. A similar trend is observed on the SSB108 dataset, where iDLDDG reaches a PCC of 0.863 compared with 0.850 for mCSM-NA. Error decomposition reveals a critical contrast between overall performance and robustness to extreme values. Although SSB108 exhibits reasonable average PCC = 0.839, its predictive performance on extreme ΔΔG values deteriorates sharply, with the PCC dropping to 0.079 for the top 10% outliers, as illustrated in Fig. 6E. DSB444 shows a comparable vulnerability, with an outlier PCC of 0.119, despite maintaining a stronger baseline performance (PCC = 0.664).

Independent testing reveals a significant performance disparity: the outliers of IDSB70 paradoxically approach perfection (PCC = 0.896), while the overall performance remains modest (PCC = 0.489). This divergence suggests systematic residue–interface prediction errors rather than random noise. Conversely, ISSB26 achieves extraordinary outlier stability (PCC = 0.994) alongside robust overall metrics (PCC = 0.750; RMSE = 1.739 kcal/mol). Collectively, these patterns demonstrate the superior adaptability of iDLDDG to SSB landscapes despite the constrained training complexity of this task. For DSBs, however, the performance plateaus reflect the high-dimensional conformational search space inherent to double-stranded DNA recognition, particularly sequence-specific groove penetration and dynamic hydrogen-bonding arrays.

#### Performance differences explained by data distribution

The predictive performance of iDLDDG is closely linked to the distribution characteristics of the underlying data. As illustrated in Fig. 6, scatter plots show strong agreement between predicted and experimental ΔΔG values along the diagonal, while histograms reveal that both follow a near-normal distribution centered between −2 and 2 kcal/mol.

Data distribution characteristics directly influence model performance. The ISSB26 dataset, which displays higher intrinsic variability, results in elevated MAE (1.519) and RMSE (1.739) values. In contrast, iDLDDG achieves robust accuracy on the more compactly distributed SSB-type data, with a cross-validation PCC of 0.863 on SSB108 and an independent test PCC of 0.707 on ISSB26. Performance on DSB data is more moderate (cross-validation PCC = 0.681, independent PCC = 0.457), reflecting its broader distributional spread.

Notably, reverse augmentation applied to MPD552 balances its distribution around a near-zero mean, amplifying the influence of outliers on RMSE and making it a sensitive error indicator. Across all evaluations, iDLDDG consistently outperforms existing methods, achieving superior PCC on both MPD552 and MPD96 (Table 3) and surpassing mCSM-NA under structure-specific training (Fig. 5). These results collectively demonstrate that iDLDDG’s performance profile is systematically shaped by the distributional properties of the data it encounters.

#### Performance comparison with different machine learning predictors

To assess the effectiveness of iDLDDG relative to conventional machine learning methods on a limited dataset, we conducted a comparative study using six widely adopted regressors. ESM2, ESM1v, and ProtTrans were employed to generate residue-level representations for each protein sequence. Specifically, for a protein of length 181, ESM2 and ESM1v each produced embeddings of size 181 × 1280, while ProtTrans generated embeddings of size 181 × 1024. To unify the feature dimensions across different models, the embeddings from each representation were independently passed through two fully connected layers, reducing their dimensionality to 181 × 256, as illustrated in Fig. 2. Finally, the three reduced feature matrices were concatenated, producing a 181 × 768 representation. These fused embeddings served as input for predicting ∆∆G under 10-fold cross-validation, using Support Vector Regression [42], Random Forest [43], Gradient Boosting [44], AdaBoost [45], Gaussian Process Regression [46], and XGBoost [47].

As summarized in Table 4, iDLDDG achieved a PCC of 0.755, with an RMSE below 1.0 and an MAE of 0.674, demonstrating a marked improvement over all conventional machine learning regressors. The baseline models, which included Support Vector Regression, Random Forest, and XGBoost, yielded PCC values ranging from 0.081 to 0.451. This performance gap demonstrates the framework’s capacity to model high-dimensional feature representations from PLMs. Detailed hyperparameters for all baseline models are listed in Table S4.

**Table 4:** Performance comparison among different predictors on MPD552 via 10-fold cross-validation.

Predictor	PCC (kcal/mol)	RMSE (kcal/mol)	MAE (kcal/mol)
Support vector regression	0.451	1.551	1.138
Random forest	0.314	1.615	1.127
Gradient boost	0.264	1.560	1.096
AdaBoost	0.242	1.512	1.081
Gaussian regression	0.246	1.424	1.035
XGBoost	0.081	1.697	1.164
**iDLDDG**	**0.755**	**0.964**	**0.674**

#### Feature visualization analysis for explaining the strong performance of iDLDDG

**Figure 7:**
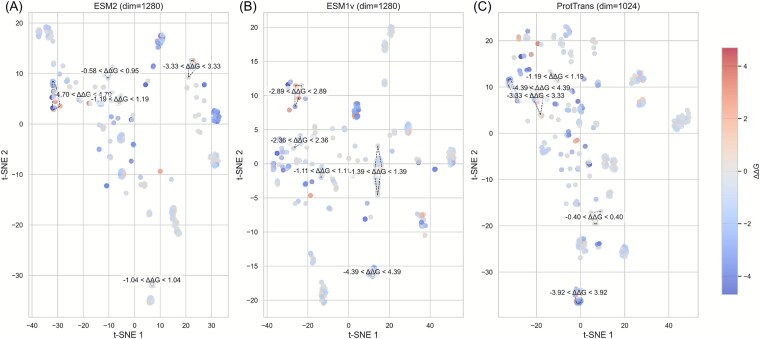
t-SNE maps of protein-based large language model embeddings for discriminating ΔΔG values. (A)–(C) ESM2, ESM1v, and ProtTrans.

To elucidate the strong performance of iDLDDG, we visualized the embeddings from ESM2, ESM1v, and ProtTrans using t-SNE [48], coloring each point by its ΔΔG value. This approach reveals how effectively each model separates stabilizing from destabilizing mutations (Fig. 7).

To analyze the clustering behaviors and discriminative strengths of the models, we applied DBSCAN [49] to the t-SNE coordinates. Parameters were set with “eps” as the median distance to the 15th nearest neighbor and “min_samples” fixed at 15. For each subplot, we selected three to five representative clusters based on their mean ΔΔG values and evenly distributed indices, ensuring a broad representation across the ΔΔG range.

As shown in Fig. 7A, the ESM2 embeddings yielded 15 clusters (eps = 2.19). The representative clusters spanned ΔΔG ranges such as −2.50 to −1.00 and 1.00 to 2.50, demonstrating clear separation between stabilizing and destabilizing mutations. As shown in Fig. 7B, the ESM1v embeddings produced 12 clusters (eps = 2.33). However, some clusters resulted in overlapping ΔΔG ranges, suggesting less distinct separation effects. In Fig. 7C, ProtTrans embeddings generated 13 clusters (eps = 3.35), capturing wider ΔΔG intervals such as −3.00 to −1.50 and 0.50 to 3.00, which indicates robust discriminative capabilities.

The clustering analysis highlights model-specific ΔΔG patterns. For example, ESM2 excels at resolving intermediate mutation effects (ΔΔG∈[−2.50, −1.00] and [1.00, 2.50]), while ProtTrans captures a broader range, including extreme values (e.g. [−3.00, −1.50] and [0.50, 3.00]), thus reflecting greater sensitivity to diverse mutational impacts. In contrast, ESM1v, with fewer clusters and some overlap, may provide specialized insights within narrower ΔΔG bands.

These distinct clustering profiles suggest that each model uncovers unique facets of the mutational landscape. Consequently, their embeddings could offer complementary perspectives for analyzing mutation effects. For additional details on the feature contributions, refer to Table 2 and Section 4.4.

### Case study

**Figure 8:**
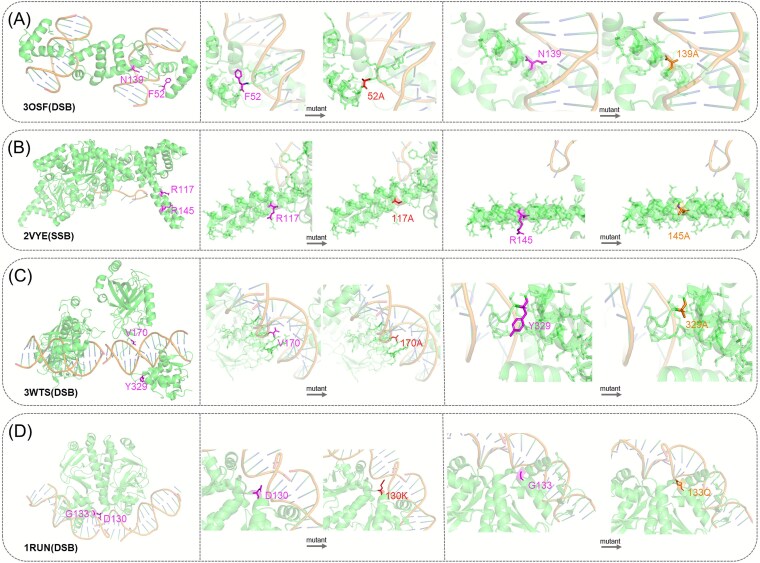
Local protein structure and mutation information. (A) PDB_ID: 3OSF (F52A, N139A). (B) PDB_ID: 2VYE (R117A, R145A). (C) PDB_ID: 3WTS (V170A, Y329A). (D) PDB_ID: 1RUN (D130K, G133Q).

We evaluate iDLDDG’s predictive performance using 10-fold cross-validation on the MPD552 dataset. Its predictions for six mutations across three proteins are compared with mCSM-NA [15] in Table 4. The analysis examines mutations that cause both substantial and minimal ΔΔG changes, assessing performance for both DSB and SSB. Figure 8 complements this analysis by illustrating mutation sites near protein–DNA interfaces, clarifying their impacts on binding affinities. ΔΔG values can be categorized to reflect their impact on protein stability. Mutations with ΔΔG greater than 2.0 kcal/mol are considered strongly destabilizing, indicating a significant reduction in protein stability. Mutations with ΔΔG between 0.5 and 2.0 kcal/mol are classified as moderately destabilizing, having a moderate effect on stability. Mutations with ΔΔG between −0.5 and 0.5 kcal/mol are regarded as negligible effect, exerting minimal influence on stability. Finally, mutations with ΔΔG less than −0.5 kcal/mol are considered stabilizing, contributing to increased protein stability.

Figure 8 displays the structures of four proteins (3OSF, 2VYE, 3WTS, and 1RUN), highlighting their specific mutations. For each mutation site near protein**–**DNA interfaces, subplots show the protein’s ribbon structure with magnified stick views of wild-type and mutant residues. As shown in Fig. 8A (3OSF), the F52A and N139A mutations are located at the interface, potentially disrupting essential DNA interactions, while Fig. 8B (2VYE) shows the R117A and R145A mutations near the interface, which are likely to influence single-stranded DNA binding. Figure 8C (3WTS) presents the V170A and Y329A mutations adjacent to a DNA molecule, suggesting their involvement in double-stranded DNA interactions. Specifically, for the 3WTS protein (Fig. 8C), the V170A mutation results in minimal binding affinity change (ΔΔG = 0.10 kcal/mol). V170A may stabilize the hydrophobic core or promote DNA binding through hydrophobic interactions. Substitution with alanine reduces side-chain volume while preserving hydrophobicity, explaining the negligible effect. Figure 8D (1RUN) primarily depicts mutations resulting in negative ΔΔG values, which correspond to stabilizing effects.

As listed in Table 5, for mutations that induce significant changes in the binding affinity (ΔΔG ≥ 1.97 kcal/mol), iDLDDG exhibits superior predictive accuracy across both DSB and SSB proteins. This performance advantage is evidenced by two interfacial mutations in the DSB protein 3OSF (Fig. 8A). For the N139A variant, experimental measurements yield ΔΔG = 2.28 kcal/mol. iDLDDG accurately predicts this change (2.31 kcal/mol), while mCSM-NA [15] generates an erroneous destabilization value of −0.266 kcal/mol. Similarly, for the F52A mutation (experimental ΔΔG = 2.81 kcal/mol), iDLDDG closely approximates the experimental value (2.703 kcal/mol), contrasting sharply with mCSM-NA’s miscalculated prediction of −1.391 kcal/mol.

**Table 5:** Performance comparison among different predictors on MPD552 when using 10-fold cross-validation.

PDB_ID	Chain	Mutation	Type	ΔΔG (kcal/mol)	iDLDDG	mCSM-NA
3OSF	A	N139A	DSBs	2.28	2.31	−0.266
3OSF	A	F52A	DSBs	2.81	2.703	−1.391
2VYE	A	R145A	SSBs	1.97	1.905	−1.350
2VYE	A	R117A	SSBs	2.00	1.862	−1.351
3WTS	A	V170A	DSBs	0.10	0.138	−0.098
3WTS	C	Y329A	DSBs	0.31	0.204	−0.402
1RUN	A	D130K	DSBs	−1.46	−1.048	0.406
1RUN	A	G133Q	DSBs	−1.24	−1.653	−0.704

Regarding the SSB-associated protein 2VYE, iDLDDG accurately predicts 1.905 kcal/mol for the R145A mutation (experimental ΔΔG = 1.97 kcal/mol) and 1.862 kcal/mol for the R117A mutation (experimental ΔΔG = 2.00 kcal/mol), both of which are near the DNA-binding interface (Fig. 8B). Conversely, mCSM-NA yields negative predictions (−1.350 kcal/mol for R145A and − 1.351 kcal/mol for R117A), failing to capture the destabilizing effects of these mutations. The structural proximity of these mutations to the DNA interface, as visualized in Fig. 8B, likely contributes to their significant impact on the binding affinity. In Fig. 8C, the mutations on protein 3WTS show negligible effects. iDLDDG predicts a ΔΔG of 0.138 kcal/mol for V170A, experimental ΔΔG is 0.10 kcal/mol, and 0.204 kcal/mol for a mutation with an experimental ΔΔG of 0.31 kcal/mol, whereas existing methods predicted −0.098 and −0.402 kcal/mol, respectively. On protein 1RUN, stabilizing mutations were predicted by iDLDDG to have ΔΔG values of −1.048 kcal/mol and −1.653 kcal/mol, compared to experimental ΔΔG values of −1.46 kcal/mol and −1.24 kcal/mol, while mCSM-NA predicted 0.406 kcal/mol and −0.704 kcal/mol.

Structural analysis of mutation sites near protein**–**DNA interfaces (Fig. 8) reveals that iDLDDG effectively captures local environmental features critical for DNA binding. Consequently, iDLDDG achieves lower prediction errors than mCSM-NA across both DSB and SSB datasets—particularly for mutations spanning high and low ΔΔG magnitudes (Table 5). These results establish iDLDDG as a robust computational tool for predicting missense mutation effects in DNA-binding proteins.

## Conclusions

Missense MPDs can disrupt key biological processes and contribute to disease by altering binding affinity. However, predicting the associated ∆∆G changes remain challenging, as current methods often fail to capture structural diversity and rely on hand-engineered features. In response to these challenges, we introduce iDLDDG, an innovative two-stage deep learning framework that leverages transformer architectures and PLM embeddings from ESM2, ESM1v, and ProtTrans. Critically, iDLDDG distinguishes DSB and SSB DNA-binding proteins to model their unique binding properties. By concentrating on mutation sites, the framework effectively captures essential contextual information from both wild-type and mutant sequences.

Furthermore, we devised an information entropy-based algorithm to determine the optimal protein sequence length, establishing 181 residues as the point at which information gain and entropy fluctuations reach equilibrium. This optimization scheme not only improves the predictive accuracy of the model but also enhances its computational efficiency and scalability. The first stage of the framework employs a transformer encoder with multi-head attention to amalgamate these features into a unified representation, which subsequently guides the ∆∆G prediction process. Compared with the existing state-of-the-art methods, iDLDDG outperforms the other methods on a variety of benchmark and independent datasets while preserving its computational efficiency.

Despite its strong performance, several limitations remain. First, the model’s accuracy decreases for outlier samples with extreme ∆∆G values. Second, the limited size of available training data constrains the generalization ability of iDLDDG—a challenge shared by most current predictive models for protein stability. Third, the current framework focuses on sequence-based features and may not fully capture complex tertiary or quaternary structural effects.

Future research could address these limitations by incorporating larger and more diverse datasets, integrating structural and biophysical information, and exploring hybrid models that combine deep learning with physics-based simulations. Additionally, experimental validation of predicted stabilizing or destabilizing mutations could further strengthen the reliability and applicability of iDLDDG in biomedical research and protein engineering.

**Key Points**

- We present a novel deep-learning method, termed iDLDDG, for predicting protein stability changes from missense mutations in DNA-binding proteins. We also demonstrate that DSBs- and SSBs-specific prediction models perform better than generalized models, which does not distinguish between protein types (DSBs and SSBs).
- We systematically analyzed the underlying relationship between input-data entropy and model performance based on information-entropy, which has a significant positive impact on model optimization of iDLDDG.
- iDLDDG integrates three discriminative protein representations including ESM2, ESM1v, and ProtTrans, which are further optimized to obtain the final feature representation. Extensive benchmarking experiments demonstrate that iDLDDG outperforms state-of-the-art methods.

## Supplementary Material

SI_iDLDDG_Final_bbag050

## Data Availability

Datasets, source codes, and relevant model checkpoints used in this study can be freely downloaded at https://github.com/xycsglas/iDLDDG for academic use.

## References

[1] Bischoff G, Hoffmann S. DNA-binding of drugs used in medicinal therapies. *Curr Med Chem* 2002;9:321–48. 10.2174/0929867023371085 11860361

[2] Ghosh G, Sharma R. Affinity-based clinical biomarkers for early disease detection. In: Mahato K, Chandra P (eds). *Biosensors for Personalized Healthcare*. Singapore: Springer, 2024; 49–68. 10.1007/978-981-97-5473-1_3

[3] Zhou H-X, Pang X. Electrostatic interactions in protein structure, folding, binding, and condensation. *Chem Rev* 2018;118:1691–741. 10.1021/acs.chemrev.7b00305 29319301 PMC5831536

[4] Wang X, Xue G, Song M et al. Molecular basis of rutin inhibition of protein disulfide isomerase (PDI) by combined in silico and experimental methods. *RSC Adv* 2018;8:18480–91. 10.1039/c8ra02683a 35541126 PMC9080521

[5] Fu X-M, Wang P, Zhu BT. Characterization of the estradiol-binding site structure of human protein disulfide isomerase (PDI). *PLoS One* 2011;6:e27185. 10.1371/journal.pone.0027185 22073283 PMC3207843

[6] Delgado J, Reche R, Cianferoni D et al. FoldX force field revisited, an improved version. *Bioinformatics* 2025;41:btaf064. 10.1093/bioinformatics/btaf064 39913370 PMC11879241

[7] Peng Y, Sun L, Jia Z et al. Predicting protein–DNA binding free energy change upon missense mutations using modified MM/PBSA approach: SAMPDI webserver. *Bioinformatics* 2018;34:779–86. 10.1093/bioinformatics/btx698 29091991 PMC6048991

[8] Velázquez-Campoy A, Ohtaka H, Nezami A et al. Isothermal titration calorimetry. *Curr Protoc Cell Biol* 2004;23:17.18. 11–24. 10.1002/0471143030.cb1708s23 18228446

[9] Hellman LM, Fried MG. Electrophoretic mobility shift assay (EMSA) for detecting protein–nucleic acid interactions. *Nat Protoc* 2007;2:1849–61. 10.1038/nprot.2007.249 17703195 PMC2757439

[10] Chai C, Xie Z, Grotewold E. SELEX (systematic evolution of ligands by EXponential enrichment), as a powerful tool for deciphering the protein–DNA interaction space. *Plant transcription factors: methods and protocols* 2011;754:249–58. 10.1007/978-1-61779-154-3_14 21720957

[11] Schymkowitz J, Borg J, Stricher F et al. The FoldX web server: An online force field. *Nucleic Acids Res* 2005;33:W382–8. 10.1093/nar/gki387 15980494 PMC1160148

[12] Van Durme J, Delgado J, Stricher F et al. A graphical interface for the FoldX forcefield. *Bioinformatics* 2011;27:1711–2. 10.1093/bioinformatics/btr254 21505037

[13] Prabakaran P, An J, Gromiha MM et al. Thermodynamic database for protein–nucleic acid interactions (ProNIT). *Bioinformatics* 2001;17:1027–34. 10.1093/bioinformatics/17.11.1027 11724731

[14] Zhang N, Chen Y, Zhao F et al. PremPDI estimates and interprets the effects of missense mutations on protein-DNA interactions. *PLoS Comput Biol* 2018;14:e1006615. 10.1371/journal.pcbi.1006615 30533007 PMC6303081

[15] Pires DE, Ascher DB. mCSM–NA: predicting the effects of mutations on protein–nucleic acids interactions. *Nucleic Acids Res* 2017;45:W241–6. 10.1093/nar/gkx236 28383703 PMC5570212

[16] Jiang Y, Liu H-F, Liu R. Systematic comparison and prediction of the effects of missense mutations on protein-DNA and protein-RNA interactions. *PLoS Comput Biol* 2021;17:e1008951. 10.1371/journal.pcbi.1008951 33872313 PMC8084330

[17] Ban C, Chung S, Park D-S et al. Detection of protein–DNA interaction with a DNA probe: distinction between single-strand and double-strand DNA–protein interaction. *Nucleic Acids Res* 2004;32:e110. 10.1093/nar/gnh109 15273279 PMC506829

[18] Kolluri R, Torrey TA, Kinniburgh AJ. A CT promoter element binding protein: definition of a double-strand and a novel single-strand DNA binding motif. *Nucleic Acids Res* 1992;20:111–6. 10.1093/nar/20.1.111 1738588 PMC310333

[19] Dickey TH, Altschuler SE, Wuttke DS. Single-stranded DNA-binding proteins: multiple domains for multiple functions. *Structure* 2013;21:1074–84. 10.1016/j.str.2013.05.013 23823326 PMC3816740

[20] Wang W, Liu J, Zhou X. Identification of single-stranded and double-stranded DNA binding proteins based on protein structure. *BMC bioinformatics* 2014;15:1–9. 10.1186/1471-2105-15-S12-S4 25474071 PMC4243121

[21] Wang W, Sun L, Zhang S et al. Analysis and prediction of single-stranded and double-stranded DNA binding proteins based on protein sequences. *BMC bioinformatics* 2017;18:1–10. 10.1186/s12859-017-1715-8 28606086 PMC5469069

[22] Guo J-T, Malik F. Single-stranded DNA binding proteins and their identification using machine learning-based approaches. *Biomolecules* 2022;12:1187. 10.3390/biom12091187 36139026 PMC9496475

[23] Gerelchuluun A. DNA damage, repair mechanisms, and chromosomal aberrations. In: Tsuboi K, Sakae T, Gerelchuluun A (eds). Proton Beam Radiotherapy: Physics and Biology. Singapore: Springer, 2020:183–208, 10.1007/978-981-13-7454-8_15

[24] Wu Y, Lu J, Kang T. Human single-stranded DNA binding proteins: guardians of genome stability. *Acta Biochim Biophys Sin* 2016;48:671–7. 10.1093/abbs/gmw044 27217471

[25] Marceau AH . Functions of single-strand DNA-binding proteins in DNA replication, recombination, and repair. In: Keck JL (ed). Single-Stranded DNA Binding Proteins: Methods and Protocols. Totowa, NJ: Humana Press, 2012:1–21, 10.1007/978-1-62703-032-8_1 22976174

[26] Maffeo C, Aksimentiev A. Molecular mechanism of DNA association with single-stranded DNA binding protein. *Nucleic Acids Res* 2017;45:12125–39. 10.1093/nar/gkx917 29059392 PMC5716091

[27] Lin Z, Akin H, Rao R et al. Evolutionary-scale prediction of atomic-level protein structure with a language model. *Science* 2023;379:1123–30. 10.1126/science.ade2574 36927031

[28] Rives A, Meier J, Sercu T et al. Biological structure and function emerge from scaling unsupervised learning to 250 million protein sequences. *Proc Natl Acad Sci* 2021;118:e2016239118. 10.1073/pnas.2016239118 33876751 PMC8053943

[29] Meier J, Rao R, Verkuil R et al. Language models enable zero-shot prediction of the effects of mutations on protein function. *Adv Neural Inf Proces Syst* 2021;34:29287–303.

[30] Elnaggar A, Heinzinger M, Dallago C et al. Prottrans: toward understanding the language of life through self-supervised learning. *IEEE Trans Pattern Anal Mach Intell* 2021;44:7112–27. 10.1109/TPAMI.2021.3095381 34232869

[31] Liu L, Xiong Y, Gao H et al. dbAMEPNI: a database of alanine mutagenic effects for protein–nucleic acid interactions. *Database* 2018;2018:bay034. 10.1093/database/bay034 29688380 PMC5887268

[32] Bank PD . Protein data bank. *Nat New Biol* 1971;233:10–1038.

[33] Steinegger M, Mirdita M, Söding J. Protein-level assembly increases protein sequence recovery from metagenomic samples manyfold. *Nat Methods* 2019;16:603–6. 10.1038/s41592-019-0437-4 31235882

[34] Steinegger M, Söding J. Clustering huge protein sequence sets in linear time. *Nat Commun* 2018;9:2542. 10.1038/s41467-018-04964-5 29959318 PMC6026198

[35] Klausen MS, Jespersen MC, Nielsen H et al. NetSurfP-2.0: improved prediction of protein structural features by integrated deep learning. *Proteins: Structure, Function, and Bioinformatics* 2019;87:520–7. 10.1002/prot.25674 30785653

[36] Consortium U . UniProt: a worldwide hub of protein knowledge. *Nucleic Acids Res* 2019;47:D506–15. 10.1093/nar/gky1049 30395287 PMC6323992

[37] Suzek BE, Wang Y, Huang H et al. UniRef clusters: a comprehensive and scalable alternative for improving sequence similarity searches. *Bioinformatics* 2015;31:926–32. 10.1093/bioinformatics/btu739 25398609 PMC4375400

[38] Devlin J, Chang M-W, Lee K et al. Bert: Pre-training of deep bidirectional transformers for language understanding. In: Burstein J, Doran C, Solorio T (eds). Proceedings of the 2019 conference of the North American chapter of the association for computational linguistics: human language technologies, volume 1 (long and short papers). Stroudsburg, PA: Association for Computational Linguistics, pp. 4171–86, 2019.

[39] Suzek BE, Huang H, McGarvey P et al. UniRef: comprehensive and non-redundant UniProt reference clusters. *Bioinformatics* 2007;23:1282–8. 10.1093/bioinformatics/btm098 17379688

[40] Zhang M, Gong C, Ge F et al. FCMSTrans: accurate prediction of disease-associated nsSNPs by utilizing multiscale convolution and deep feature combination within a transformer framework. *J Chem Inf Model* 2024;64:1394–406. 10.1021/acs.jcim.3c02025 38349747

[41] Li C-F, Yan Z, Ge F et al. TransABseq: a two-stage approach for predicting antigen–antibody binding affinity changes upon mutation based on protein sequences. *J Chem Inf Model* 2025;65:5188–204. 10.1021/acs.jcim.5c00478 40354482

[42] Awad M, Khanna R, Awad M et al. Support vector regression. *Efficient learning machines: Theories, concepts, and applications for engineers and system designers* 2015;1:67–80. 10.1007/978-1-4302-5990-9_4

[43] Breiman L . Random forests. *Mach Learn* 2001;45:5–32. 10.1023/A:1010933404324

[44] Friedman JH . Stochastic gradient boosting. *Computational statistics & data analysis* 2002;38:367–78. 10.1016/S0167-9473(01)00065-2

[45] Schapire RE . Explaining adaboost. In: Schölkopf B, Luo Z, Vovk V (eds). *Empirical Inference: Festschrift in Honor of Vladimir N. Vapnik*. Berlin, Heidelberg: Springer, 2013;37–52. 10.1007/978-3-642-41136-6_5

[46] Williams C, Rasmussen C. Gaussian processes for regression. In: Touretzky D, Mozer MC, Hasselmo M (eds). Advances in Neural Information Processing Systems. **Vol. 8**, Cambridge, MA: MIT Press, 1995.

[47] Chen T, Guestrin C. Xgboost: A scalable tree boosting system. In: Krishnapuram B, Shah M (eds). Proceedings of the 22nd acm sigkdd international conference on knowledge discovery and data mining. New York, NY: Association for Computing Machinery, 2016; 785–794.

[48] Van der Maaten L, Hinton G. Visualizing data using t-SNE. *J Mach Learn Res* 2008;9:2579–605.

[49] Schubert E, Sander J, Ester M et al. DBSCAN revisited, revisited: why and how you should (still) use DBSCAN. *ACM Transactions on Database Systems (TODS)* 2017;42:1–21. 10.1145/3068335

